# The other side of the pandemic: effects of Coronavirus crisis among student’s mental health

**DOI:** 10.1192/j.eurpsy.2023.1638

**Published:** 2023-07-19

**Authors:** A. H. I. Abu Shehab, L. Luca, D. C. Voinescu, I.-A. Ciureanu, I. Udriştoiu, S. Trifu, A. Ciubară

**Affiliations:** 1 Psychiatry, “Elisabeta Doamna” psychiatry hospital, Galati; 2 Psychology, University of Medicine and Pharmacy “Grigore T. Popa”, Iasi; 3 Rheumatology, „Dunărea de Jos” University of medicine, Galati; 4 Medical informatics department, University of Medicine and Pharmacy “Grigore T. Popa”, Iasi; 5 Psychiatry, University of Medicine and Pharmacy of Craiova, Craiova; 6 Psychiatry, Carol Davila University of Medicine and Pharmacy, Bucharest; 7 Psychiatry, „Dunărea de Jos” University of medicine, Galati, Romania

## Abstract

**Introduction:**

The outbreak of COVID-19 has long-term negative effects on mental health. This study shows the negative mental health effects of studying under pandemic limits involving distance learning and social isolation.

**Objectives:**

The specialized studies carried out after the emergence of the Coronavirus revealed the impact of the measures implemented during the period of restrictions and after the outbreak of the pandemic, as well as the way in which these measures were felt by the general population.

**Methods:**

Qualitative analysis of students’ answers regarding the stress felt after the outbreak of the pandemic.

**Results:**

Social and individual anxiety remains a subject of investigation among female students, who are in the process of emotional maturation and professional training.

**Conclusions:**

Students remain a vulnerable population category, in the conditions in which society is in full post-pandemic adaptation process.

**Disclosure of Interest:**

None Declared

